# Fecal luminal factors from patients with irritable bowel syndrome induce distinct gene expression of colonoids

**DOI:** 10.1111/nmo.14390

**Published:** 2022-04-29

**Authors:** Cristina Iribarren, Sofia Nordlander, Johanna Sundin, Stefan Isaksson, Otto Savolainen, Hans Törnblom, Maria K. Magnusson, Magnus Simrén, Lena Öhman

**Affiliations:** ^1^ Department of Microbiology and Immunology Institute of Biomedicine Sahlgrenska Academy University of Gothenburg Gothenburg Sweden; ^2^ Department of Molecular and Clinical Medicine Institute of Medicine Sahlgrenska Academy University of Gothenburg Gothenburg Sweden; ^3^ Chalmers Mass Spectrometry Infrastructure Department of Biology and Biological Engineering Chalmers University of Technology Gothenburg Sweden; ^4^ Center for Functional GI and Motility Disorders University of North Carolina Chapel Hill North Carolina USA

**Keywords:** colonoids, epithelial barrier, host‐microbiota cross talk, intestinal microenvironment, irritable bowel syndrome

## Abstract

**Background:**

Alteration of the host‐microbiota cross talk at the intestinal barrier may participate in the pathophysiology of irritable bowel syndrome (IBS). Therefore, we aimed to determine effects of fecal luminal factors from IBS patients on the colonic epithelium using colonoids.

**Methods:**

Colon‐derived organoid monolayers, colonoids, generated from a healthy subject, underwent stimulation with fecal supernatants from healthy subjects and IBS patients with predominant diarrhea, phosphate‐buffered saline (PBS), or lipopolysaccharide (LPS). Cytokines in cell cultures and fecal LPS were measured by ELISA and mRNA gene expression of monolayers was analyzed using Qiagen RT^2^ Profiler PCR Arrays. The fecal microbiota profile was determined by the GA‐map^™^ dysbiosis test and the fecal metabolite profile was analyzed by untargeted liquid chromatography/mass spectrometry.

**Key results:**

Colonoid monolayers stimulated with fecal supernatants from healthy subjects (*n* = 7), PBS (*n* = 4) or LPS (*n* = 3) presented distinct gene expression profiles, with some overlap (R^2^Y = 0.70, Q^2 ^= 0.43). Addition of fecal supernatants from healthy subjects and IBS patients (*n* = 9) gave rise to different gene expression profiles of the colonoid monolayers (R^2^Y = 0.79, Q^2 ^= 0.64). Genes (*n* = 22) related to immune response (CD1D, TLR5) and barrier integrity (CLDN15, DSC2) contributed to the separation. Levels of proinflammatory cytokines in colonoid monolayer cultures were comparable when stimulated with fecal supernatants from either donor types. Fecal microbiota and metabolite profiles, but not LPS content, differed between the study groups.

**Conclusions:**

Fecal luminal factors from IBS patients induce a distinct colonic epithelial gene expression, potentially reflecting the disease pathophysiology. The culture of colonoids from healthy subjects with fecal supernatants from IBS patients may facilitate the exploration of IBS related intestinal micro‐environmental and barrier interactions.

## Graphical abstract

Colonoids from healthy subject cultured with fecal supernatants from IBS patients may facilitate the exploration of disease‐related intestinal micro‐environmental and barrier interactions.

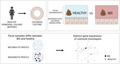

## 1 INTRODUCTION

The intestinal epithelial barrier is composed of a single layer of cells, which allows the absorption of microbial and dietary metabolites and limits the access of harmful antigens and commensal bacteria to the underlying tissues.^1^, ^2^ Therefore, the loss of homeostatic host‐microbiota cross talk may compromise the integrity of the intestinal epithelial barrier and lead to gastrointestinal diseases such as irritable bowel syndrome (IBS).^1^, ^3^ Indeed, there are reports of an impaired intestinal epithelial barrier,^4^, ^5^ altered mucosal expression of antibacterial genes^6^ and imbalance of microbiota composition^7^, ^8^ in at least subgroups of patients with IBS. Furthermore, it has been proposed that IBS patients with diarrhea have an altered fecal metabolite profile compared to healthy subjects.^9^, ^10^, ^11^

Hitherto, functional and structural properties of the intestinal epithelial barrier of patients with IBS have been explored using cell lines or tissue samples. Stimulation with plasma, soluble mediators from colonic biopsies as well as luminal proteases from patients with IBS negatively influenced the integrity of the colon cancer derived Caco‐2 cell line.^12^, ^13^, ^14^ Further, intestinal barrier permeability in IBS has been functionally addressed ex vivo by Ussing chamber experiments and characterized by the expression of adhesion proteins in short‐term cultures of intestinal biopsies.^13^, ^15^ However, the often used Caco‐2 cell line, an immortalized line originally derived from a single donor, provide a limited and simplistic physiological representation of the gut,^16^ whereas access to primary intestinal epithelial cells and biopsies may be limited, and when available only suitable for short‐term experiments.^17^

During recent years, intestinal organoids have evolved as an attractive alternative for studying physiology, cell‐cell or microbe‐cell interactions. Compared to traditional in vitro methods, intestinal organoids provide a cell culture system more accurately mimicking the intestinal epithelium^18^, ^19^ with long‐term viability.^20^ Organoids generated from tissue‐resident stem cells under specific culture conditions resemble the organ from which they have been derived and after which they are named.^21^ Organoids from colonic tissue, termed as colonoids, grow as spheres with the apical surface facing inwards^19^ and can be seeded as monolayers for stimulation assays.^18^, ^19^ Traditionally, organoids are developed from the study subject (patients/healthy) of interest.^22^ However, the impact of the local intestinal microenvironment for intestinal epithelial barrier configuration and function is lost in this set‐up. Hence, model systems to specifically study interactions between the local intestinal microenvironment and the epithelium, providing possibilities to better understand the complex pathophysiology of IBS, is much warranted.

We hypothesized that supernatants from fecal samples, containing microbial ligands, metabolites, and other luminal factors, provide stimuli similar to that present in the intestinal lumen, which will reproduce the intestinal microenvironment of the donor who provided the fecal material. Therefore, with the aim to identify IBS‐specific regulation of colonic epithelium, we determined the effects of fecal luminal factors from IBS patients in colonoids established from a healthy donor.

## 2 MATERIAL AND METHODS

### 2.1 Study subjects and sample collection

Patients with IBS with predominant diarrhea and high severity of symptoms, based on the Bristol Stool Form scale^23^ and IBS Severity Scoring System (IBS‐SSS),^24^ respectively, were selected among the baseline data of participants from a previously reported intervention study.^25^ Patients were diagnosed according to the Rome IV criteria.^26^ The healthy subjects were randomly selected from a previous study^8^ and had no previous or current history of gastrointestinal diseases.^8^ All study participants fulfilled the inclusion criteria described in Data S1. Prior to visiting the clinic, study subjects collected fecal samples at home and kept them in the freezer until transportation. Samples were then stored on site at −80°C until preparation of fecal supernatants. Further, a healthy subject provided sigmoid colonic biopsies (25–35 cm proximal from the anus), without prior bowel preparation, using standard biopsy forceps (Olympus, 3.3 mm). All subjects gave written informed consent prior to participation in the corresponding studies.^8^, ^25^ The protocols were approved by the Regional Ethical Review Board at the University of Gothenburg (Reg. No. 266–16, 18 April 2016; Reg. No. 548–16, 4 July 2016; and Reg. No. 988–14, 9 February 2015).

### 2.2 Preparation of fecal supernatants

Feces were weighed and dissolved in two weight volumes of ice‐cold phosphate‐buffered saline (PBS), prior centrifugation for 10 min at 1,600 ***g***. The liquid phase was then ultra‐centrifuged at 35,000 ***g*** for 2 h at 4°C. The collected fecal supernatant was stored at −80°C until use.

### 2.3 Establishment of human colonoid cultures

Colonic crypts were isolated from sigmoidal colonic biopsies from a healthy subject following protocols established elsewhere^27^ with minor modifications. Briefly, first crypts and later colonoids, were maintained embedded in 40 µl solid Matrigel^®^ Matrix (Corning^®^) containing 1 µM Jagged‐1 peptide (AnaSpec), in 24‐well plates (Nuclon^™^ Delta Surface, Thermo Fisher Scientific) and were cultured in expansion medium as described previously.^27^ The expansion medium was complete medium (Advanced Dulbecco's modified Eagle medium (DMEM)/Ham's F‐12, 100 U penicillin/streptomycin, 10 mM HEPES and 0.2 mM GlutaMAX (purchased from Gibco^®^, Life Technologies^™^) supplemented with 1× B27 supplement, 1× N2 supplement, 50 ng/ml human epidermal growth factor (purchased from Gibco^®^), 1 μg/ml [Leu‐15] Gastrin (AnaSpec), 500 nM A 83–01, 10 μM SB202190, 10 nM prostaglandin E2 and 1mM N‐acetylcysteine (from Sigma‐Aldrich), 100 μg/ml Primocin (InvivoGen), 10 μM CHIR99021 (Sigma‐Aldrich) and 10 μM Y‐27632 dihydrochloride (Tocris).^27^ In addition, the media contained 50% Wnt3A conditioned medium, 20% R‐spondin conditioned medium and 10% Noggin conditioned medium. The cell lines used to supply Wnt3A and Noggin were kind gifts from Professor Hans Clevers (Hubrecht Institute); the Cultrex^®^ R‐spondin1 cell line was purchased from Trevigen, Inc. The expansion medium, without CHIR99021 and Y‐27632,^27^ was replaced every 2–3 days. Colonoids were maintained at 37°C, 5% CO_2_. When denser colonoid cultures had been established, colonoids were passaged every 7 days. All passages were performed by using Corning Cell Recovery Solution (Corning^®^) and dissolving the Matrigel on ice similar to reference,^27^ without using an orbital shaker. The dissolved matrigel was collected into basal media (DMEM/Ham's F‐12 with 1× Glutamax, 10 mM HEPES and 10% heat‐inactivated fetal bovine serum (FBS)). The colonoid structures were disrupted by pipetting and approximately 50 colonoids were seeded in each well. After each passage, cells were cultured in expansion media as described above.^27^

### 2.4 Establishment of colonoid monolayers

Colonoid monolayers were established from colonoid cultures harvested after 7 days. To establish a monolayer, approximately 200 colonoids were completely disaggregated with the help of a 27‐gauge needle. Approximately 100 μl of cell suspension in IntestiCult^™^ Organoid Growth medium (Human) (Stemcell^™^ Technologies) with 10 μM Y‐27632 and 100 μg/ml Primocin (InvivoGen) were seeded on permeable polyester filter insert (Corning Transwell, pore size 0.4 μM) coated with 10 μg/ml human collagen IV (Sigma‐Aldrich) solution, in 24‐well plates.^27^ An additional 50 and 600 μl of IntestiCult^™^ Organoid Growth medium with Y‐27632 and Primocin was added to the transwell filter and the well, respectively, and kept in culture for 3 days. Then, expansion medium was substituted with differentiation medium, where 50% v/v IntestiCult (Component B) was substituted by DMEM/Ham's F‐12. The differentiation medium was replaced with fresh differentiation medium after 2 days and colonoid monolayers were then kept in culture for one additional day to reach 3 days of differentiation.

### 2.5 Stimulation of colonoid monolayers with fecal supernatants

**FIGURE 1:**
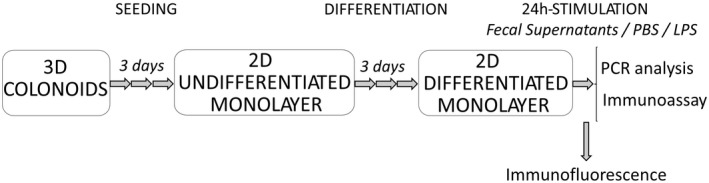
Schematic diagram of the experimental setup. Three‐dimensional (3D) colonoids generated from biopsies from a healthy subject were seeded as monolayers on transwell membranes and grown for 3 days until confluence. After 3 days of differentiation, colonoid monolayers were fixed for immunofluorescence staining or stimulated with fecal supernatants for 24 h. Stimulation with phosphate‐buffered saline (PBS) and lipopolysaccharide (LPS) were used as negative and positive controls, respectively. After stimulation, RNA was isolated for gene expression analysis and culture supernatants collected for detection of cytokines. 3D, 3‐dimensional; 2D, 2‐dimensional; RNA, ribonucleic acids

The experimental design is schematically shown in Figure 1. Differentiated colonoid monolayers were stimulated for 24 h at 37°C with fecal supernatant from either healthy subjects or IBS patients to both transwell compartments at a concentration of 1:100. For negative controls, an equal volume of PBS was added to generate non‐stimulated monolayers. Colonoid monolayers stimulated with lipopolysaccharide (LPS, InvivoGen), diluted in DMEM/Ham's F‐12 and added to a final concentration of 100 ng/ml,^28^ were used as proinflammatory, positive controls.

### 2.6 Immunofluorescence and imaging

Triple immunofluorescence staining was performed against muc‐2, phospho‐ezrin or wheat germ agglutinin, in combination with phalloidin and Hoechst, in filter inserts in a 24‐well plate as previously described.^18^ Rabbit anti‐muc2 IgG (dilution 1:400), rabbit anti‐phospho‐Ezrin IgG (dilution 1:200) were used as primary antibodies (Abcam), while the secondary antibody was anti‐rabbit Alexa 488 (dilution 1:200, Life Technologies). Probes included Phalloidin‐647 (dilution 1:500, Abcam) and wheat germ agglutinin‐488 (dilution 1:500, Thermo Fisher Scientific). Hoechst 33342 (dilution 1:10,000, Thermo Fisher Scientific) was used to stain the nucleus of cells at a final step. Washes with PBS buffer followed every step described.^18^ Finally, stained filter inserts were mounted with ProLong Gold Antifade Mountant (Thermo Fisher Scientific) on a glass slide. The preparations were kept sealed at 4°C until visualization. Images of stained monolayers were acquired using 20× objective and the same acquisition settings on a LSM 700 inverted confocal microscope (Carl Zeiss) using Zen 2012 SP5 (Black edition. Version 14.0.3.201) imaging software. Confocal images were processed using the software Zen 3.0 2019 (Blue version) and Fiji (ImageJ, Version 1.52p). Changes in brightness/contrast and reduction of background noise were applied to emphasize the qualitative analysis of the images. Images of the human healthy colonoids during culture were taken with Leica DM IL LED Inverted Microscope with ICC50 HD Camera (Type: 11 090 137 001) using 4× objective.

### 2.7 Gene expression analysis

The material from 2 to 3 monolayers, which underwent the same stimulus, was pooled prior to RNA extraction. Briefly, total RNA was isolated with the Reliaprep RNA Cell Miniprep System (Promega) following the manufacturer's instructions. For reverse‐transcription of RNA into complementary DNA synthesis (cDNA), RT^2^ First Strand Kit (Qiagen) was used. Following manufacturer's instructions, both cDNA and RT^2^ SYBR^®^ Green qPCR Mastermix (Qiagen) were used on a custom RT^2^ Profiler PCR array (Qiagen) targeting 86 genes related to antibacterial and inflammatory response, and epithelial barrier integrity. PCR arrays were analyzed in a QuantStudio 12K Real‐Time PCR System (Applied Biosystems^™^, Life Technologies). CT values were removed if *undetermined*; CT > 36 was adjusted to CT = 38. Genes were excluded from analysis if >60% of samples had missing or very high CT values (*n* = 11). Gene expression was calculated as 2^−∆CT^ (2^−(CT Target gene−CT Housekeeping gene)^ and the average of the housekeeping genes ACTB, B2M, GAPDH, HPRT1 and RPLP0 was used for normalization. A complete list of the genes targeted in the custom array is shown in Data S1.

### 2.8 Measurement of cytokines and LPS

Levels of IL‐1β, TNF‐α and IL‐8 in culture supernatants were measured using V‐PLEX custom human biomarker plate from MSD^®^ Multi‐Spot Assay System (Meso Scale Diagnostic) following the manufacturer's instructions. Culture supernatants obtained from both transwell compartments were evaluated. LPS levels were measured in fecal supernatants using the LPS ELISA kit (Aviva System Biology), following the manufacturer's instructions.

### 2.9 Untargeted liquid chromatography‐mass spectrometry analysis

The luminal factors of fecal supernatants were analyzed at Chalmers Mass Spectrometry Infrastructure using a non‐targeted liquid chromatography–mass spectrometry (LC‐MS) approach. Briefly, the analyses were carried out using a reversed‐phase chromatography (RP) and hydrophilic interaction chromatography (HILIC) with positive and negative electrospray ionization polarities, as described elsewhere.^29^ The samples of each study group were analyzed in separate batches, which included its own quality control samples. The analytical workflow named “notame”, described in,^30^ was used to pre‐process the acquired data and included drift correction within and between batches, data imputation using *missForest* R package and clustering of features to remove weak and repeated features.^30^ Log_10_ transformation was applied prior between‐batch‐correction to reduce possible batch effects caused by the instrument.

### 2.10 Fecal microbiota DNA analysis

The microbiota profile was determined at Genetic Analysis AS using the GA‐map^™^ dysbiosis test. In short, total bacterial genomic DNA is extracted with magnetic beads from homogenized and mechanically disrupted fecal samples. PCR is used to amplify the 16S rRNA gene (V3‐V9 regions) followed by probe labeling. The DNA probes, labeled with nucleotides, hybridize to complementary probes coupled to magnetic beads, that allow signal detection by BioCode 1000A 128‐Plex Analyzer (Applied BioCode).^31^ Fifty‐four DNA probes allow targeting ≥300 bacteria belonging to different taxonomic levels. This test generates as a result a bacterial profile based on fecal bacterial abundance (or probe signal intensity).^31^

### 2.11 Data analysis

Multivariate data analyses were used to evaluate the relationship between the different study groups (Y‐variables) and gene expression (X‐variables). Principal Component Analysis (PCA) was performed to recognize clustering in the observations based on the gene expression (X‐variables). Orthogonal Partial Least‐Squares‐Discriminant Analyses (OPLS‐DA) were performed to visualize and identify correlations between selected Y‐variables and X‐variables. R (version 3.6.2) and SIMCA^®^ software (version 15.0.2, MKS Umetrics AB) were used for these purposes. The quality of the OPLS‐DA was determined by the parameters of R^2^Y and Q^2^, indicating discrimination and predictability, respectively. R^2^Y values ≥0.5 correspond to good fit (max. R^2^Y = 1). Q^2^ values ≥0.4, but no more than 0.3 away than R^2^Y, are considered adequate for biological variables. Univariate analyses were applied to identify differences between specific variables. The statistical tests performed (parametric or non‐parametric) were based on the distribution of the data determined by Shapiro‐Wilk tests and histograms. *p* < 0.05 were considered statistically significant. More details can be found in Data S1.

## 3 RESULTS

### 3.1 Demographic information

In total, 7 healthy subjects (5 females) and 9 IBS patients (6 females) with predominant diarrhea provided fecal samples from which supernatants were prepared. The IBS patients were older than the healthy subjects (31 (26–66) vs. 22 (20–36) years), *p *< 0.05), but did not differ with regards to sex (healthy: 5 females, 2 males vs. IBS: 6 females, 3 males) or body mass index (25.4 (3.7) vs. 21.6 (2.5) kg/m^2^). For colonoid culture, a healthy 32‐year‐old female with body mass index of 22.0 kg/m^2^ provided colonic biopsies.

### 3.2 Establishment and characterization of colonoid monolayers

**FIGURE 2:**
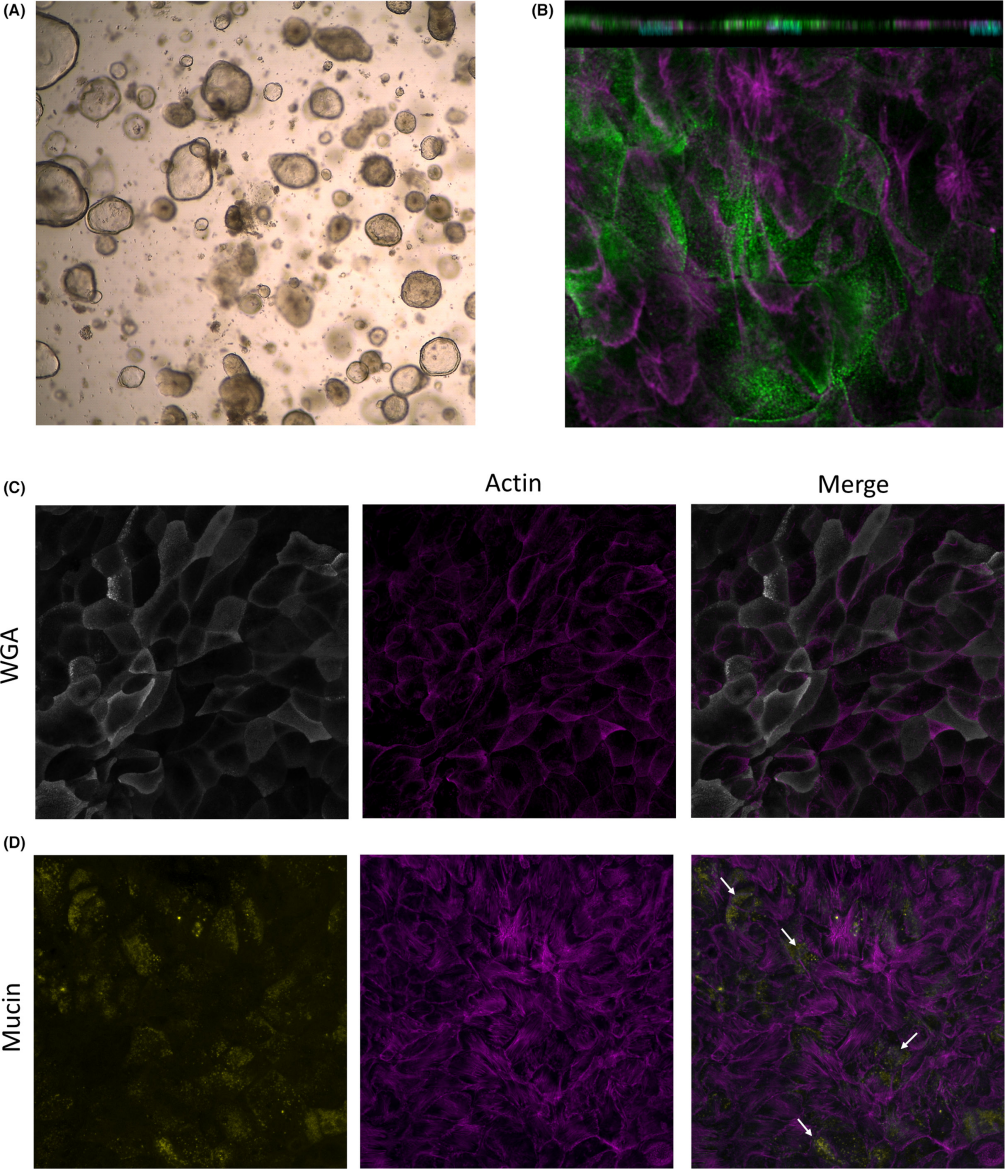
Characterization of differentiated colonoid monolayers. Colonoids obtained from colonic biopsies from a healthy subject were seeded as 2D‐monolayers and differentiated. (A) Image from light microscopy of BME‐suspended 3D colonoids; 4× magnification. (B) Polarized apical surface of cells defined by phospho‐ezrin (green) in differentiated monolayers. Orthogonal view (XZ projection), top panel. Top view (XY projection), bottom panel. (C) Glycocalyx‐rich cell membrane (wheat germ agglutinin, WGA in gray) and (D) mucin‐producing goblet cells (mucin in yellow) in differentiated colonoid monolayers. Mature microvilli and cell membrane borders on the apical surface were identified by actin expression (magenta). Nuclei are shown in cyan. Images were acquired with (A) Leica DM IL LED Inverted Microscope with ICC50 HD Camera and (B–D) LSM700 inverted confocal microscope; 20× magnification. BME, basement membrane extract

The cultures of colonoids comprised spheres with relatively thin walls and a dark core (“lumen”) containing debris. Colonoids in varying sizes were located in different focal planes throughout the extracellular matrix that supported their growth (Figure 2A). The colonoid structures were established as a monolayer comprising polarized, organized, and differentiated intestinal epithelial cells expressing phospho‐ezrin and displaying a glycocalyx (Figure 2B,C). Filamentous actin staining identified epithelial cells tightly connected by junctional complexes (Figure 2B–D). Moreover, the differentiated colonoid monolayers contained dispersed mature mucin‐producing goblet cells (Figure 2D).

### 3.3 Addition of fecal supernatants from healthy subjects modifies gene expression profile of colonoid monolayers

**FIGURE 3:**
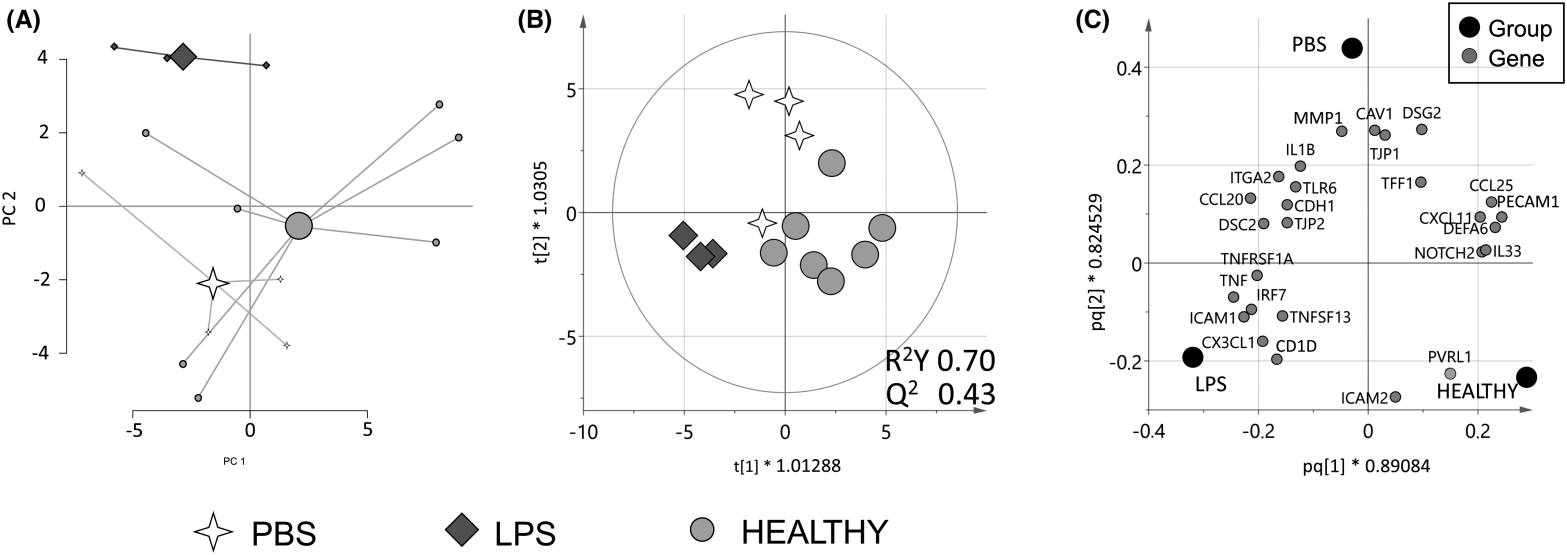
Gene expression of colonoid monolayers stimulated with fecal supernatants from healthy subjects or control stimuli. Colonoid monolayers were stimulated for 24 h with fecal supernatants from healthy subjects (*n* = 7, light gray dots), LPS (*n* = 3, dark gray diamonds) or PBS (*n* = 4, white 4‐point stars). Gene expression was analyzed by PCR arrays for genes involved in antibacterial and inflammatory response and barrier function. (A) A principal component analysis (PCA) plot based on 75 expressed genes (11 unique genes excluded as they were below detection limit). (B) Score scatter plot and (C) loading scatter plot from an orthogonal partial least squares‐discriminant analysis (OPLS‐DA) using a variable influence on projection (VIP) cut‐off >1.1. HEALTHY, colonoid monolayers stimulated with fecal supernatants from healthy subjects; LPS, lipopolysaccharide‐stimulated colonoid monolayers; PBS, PBS‐stimulated colonoid monolayers

The addition of fecal supernatants from healthy subjects, LPS or PBS to the monolayers resulted in distinct clusters, although with some overlap, based on gene expression (Figure 3A). The three different culture conditions gave rise to distinct gene expression profiles and 27 out of the 75 genes contributed to the separation between the groups (Figure 3B,C). The expression of CCL20, DSC2 and ICAM1 was lower in monolayers stimulated with fecal supernatants from healthy subjects compared to LPS‐stimulated monolayers (Figure 3C, Table S1). In contrast, the expression of PECAM1 was higher in monolayers stimulated with fecal supernatants from healthy subjects compared to LPS‐stimulated monolayers. Further, the expression of PVRL1 was higher in monolayers stimulated with fecal supernatants from healthy subjects compared to PBS‐stimulated monolayers (Figure 3C, Table S1).

### 3.4 Fecal supernatants from IBS patients induce a distinct gene expression of colonoid monolayers

**FIGURE 4:**
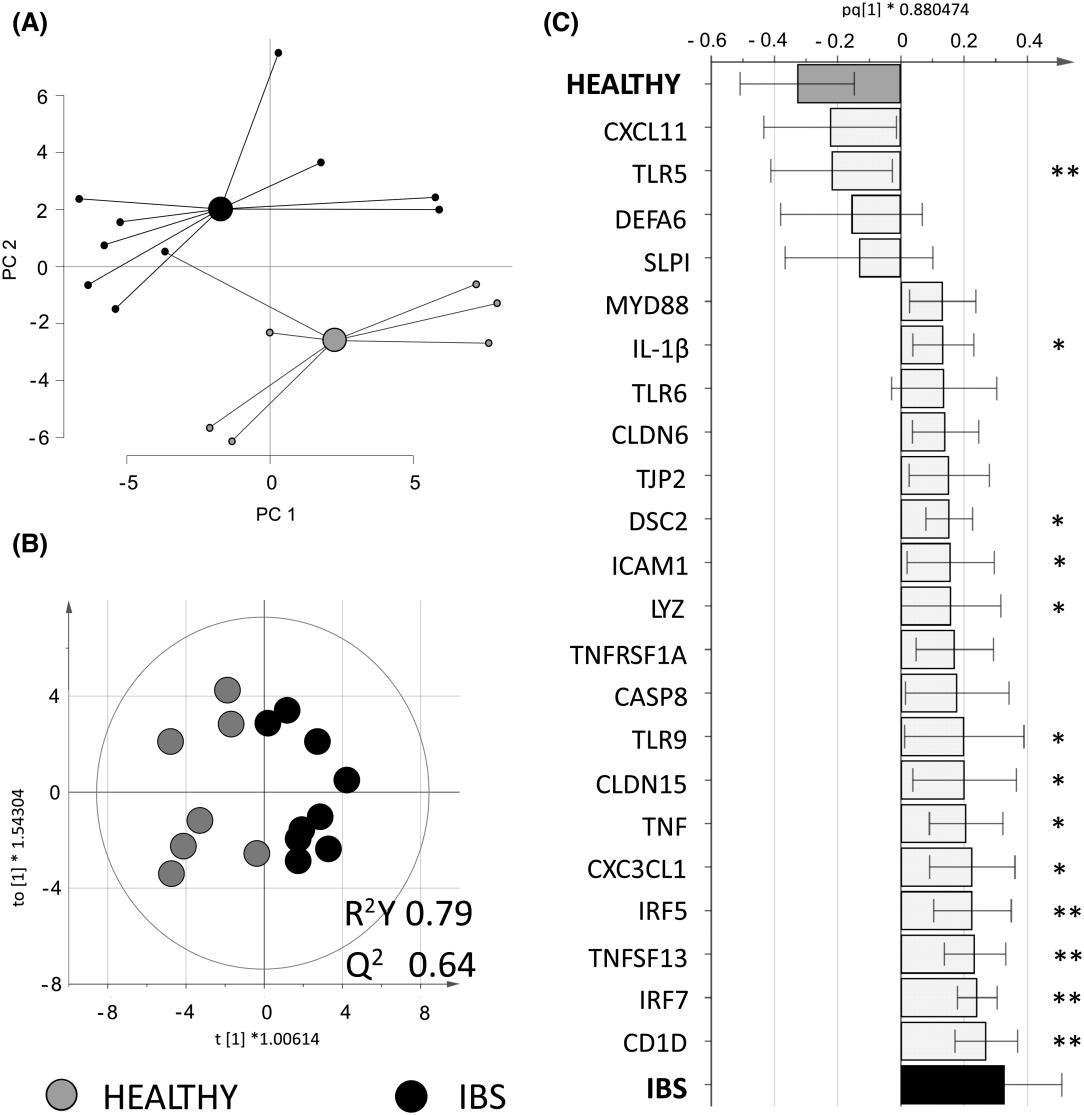
Gene expression of colonoid monolayers stimulated with fecal supernatants from healthy subjects or IBS patients. Colonoid monolayers were stimulated for 24 h with fecal supernatants from healthy subjects (*n* = 7 gray dots) or IBS patients (*n* = 9 black dots). Gene expression was analyzed by PCR arrays for genes involved in antibacterial and inflammatory response and barrier function. (A) A principal component analysis (PCA) based on 75 genes (11 unique genes excluded as they were below detection limit). (B) Score scatter plot and (C) loading column plot from an orthogonal partial least squares‐discriminant analysis (OPLS‐DA) using a VIP cut‐off >1.15. HEALTHY, colonoid monolayers stimulated with fecal supernatants from healthy subjects; IBS, colonoid monolayers stimulated with fecal supernatants from IBS patients. Between group‐comparisons were tested with Mann‐Whitney *U* test; asterisks identify statistically significant *p* values: *p < 0.05; **p < 0.01

**FIGURE 5:**
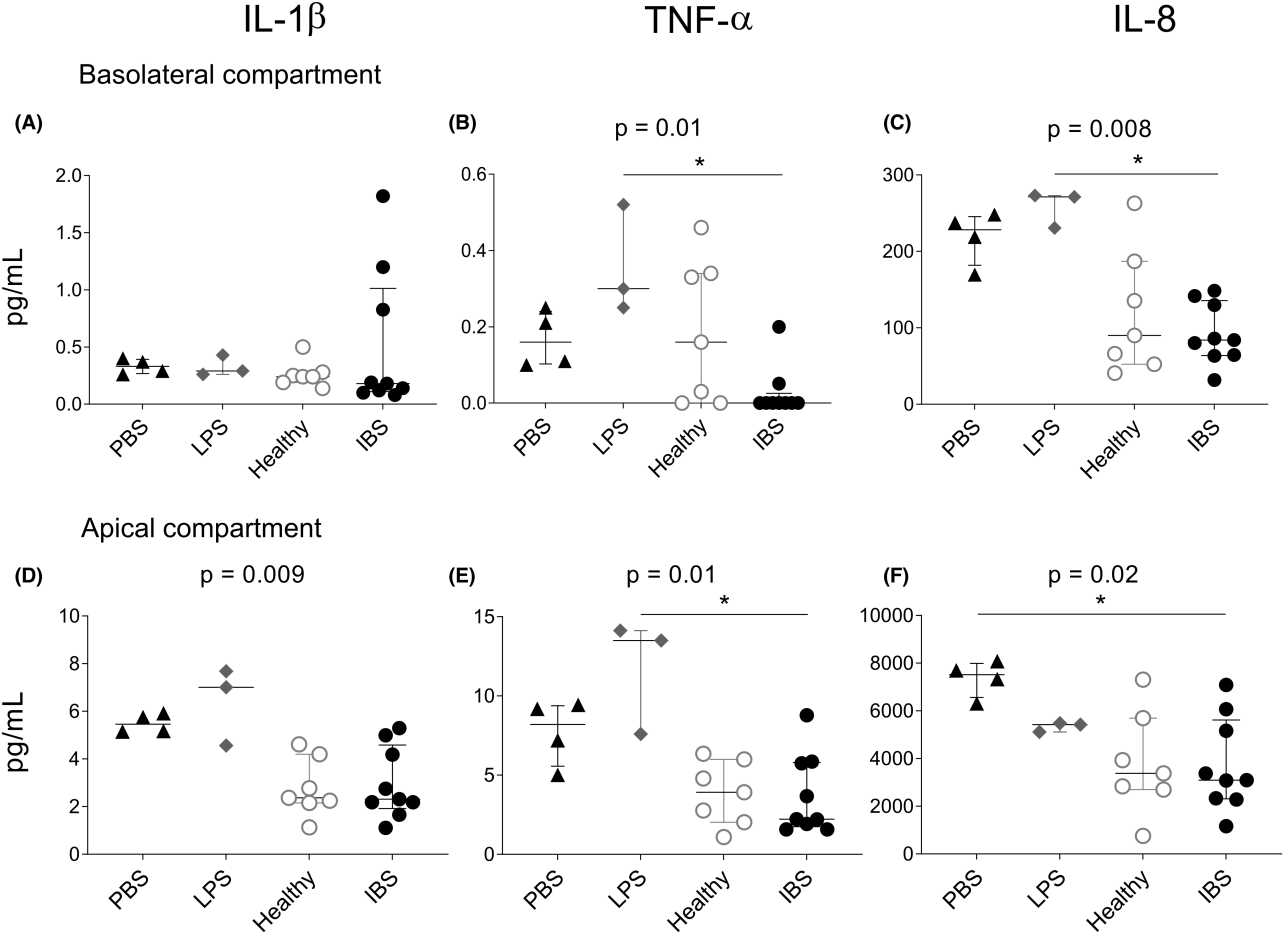
Cytokine secretion from colonoid monolayers stimulated fecal supernatants from healthy subjects or IBS patients or control stimuli. Colonoid monolayers were stimulated for 24 h with PBS (*n* = 4, black triangles), LPS (*n* = 3, gray diamonds), fecal supernatants from healthy subjects (*n* = 7 gray dots) or IBS patients (*n* = 9 black dots). Cytokine levels in culture supernatants were analyzed by MSD^®^ Multi‐Spot Assay system. (A–C) Levels in the basolateral compartment and (D–F) in the apical compartment of IL‐1β (A,D), TNF‐α (B,E) and IL‐8 (C,F). PBS, PBS‐stimulated colonoid monolayers. HEALTHY, colonoid monolayers stimulated with fecal supernatants from healthy subjects; IBS, colonoid monolayers stimulated with fecal supernatants from IBS patients; LPS, lipopolysaccharide‐stimulated colonoid monolayers; PBS, phosphate‐buffered saline‐stimulated colonoid monolayers. Cytokine concentrations (pg/ml) are shown as median (interquartile range) of two independent stimulation experiments. Between group‐comparisons were performed using the Kruskal–Wallis test with Dunn's correction; asterisks identify statistically significant *p* values: *p < 0.05

Next, we compared the effects of fecal supernatants from healthy subjects and patients with IBS on gene expression of colonoid monolayers. Addition of fecal supernatants from the two study groups to colonoid monolayers induced distinct gene expression clusters with only minor overlap (Figure 4A). In total, 22 out of the 75 genes were differently regulated by the addition of fecal supernatants from the two study groups (Figure 4B). Genes related to immune response such as CD1D, IRF7, TNFSF13, IRF5, TLR9, LYZ, ICAM1 and CX3CL1 showed higher expression in colonoid monolayers stimulated with fecal supernatants from IBS patients, while TLR5 expression was higher in colonoid monolayers stimulated with fecal supernatants from healthy subjects (Figure 4C). Also, the addition of fecal supernatants from IBS patients upregulated the gene expression of the pro‐inflammatory cytokines IL‐1β and TNF‐α and enhanced the expression of genes related to maintenance of epithelial integrity, such as DSC2, CLDN15 and TJP2 (Figure 4C). The different gene regulation of the colonoid monolayers was not explained by the fecal supernatants’ content of LPS, which was similar in both study groups (healthy subjects; 0.12 (0.01–0.55) vs. IBS patients; 0.15 (0.02–0.89) mg/ml), *p* > 0.05), nor influenced by age or sex (Figure S1). Moreover, the secreted levels of IL‐1β, TNF‐α or IL‐8 did not differ in colonoid monolayers stimulated with fecal supernatants from healthy subjects and IBS patients in either the apical or basal compartments (Figure 5). Overall, fecal supernatants caused a lower secretion of cytokines when compared to the control stimuli, that is, PBS and LPS, and cytokines were detected at very low levels in the basolateral compartment compared to the apical compartment (Figure 5).

### 3.5 The fecal microbiota and metabolite profiles distinguish healthy subjects and IBS patients

**FIGURE 6:**
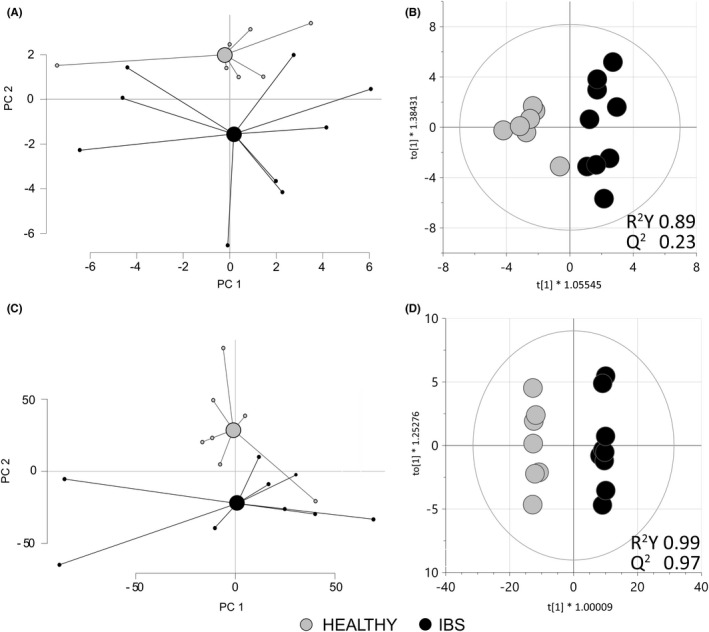
Fecal microbiota and metabolite profiles from healthy subjects and IBS patients. Fecal samples were analyzed by GA‐map^™^ dysbiosis test while fecal supernatants were analyzed by untargeted liquid chromatography/mass spectrometry analysis from healthy subjects (*n* = 7 gray dots) or IBS patients (*n* = 9 black dots). (A) A principal component analysis (PCA) based on the 54 bacterial taxa determined in fecal samples. (B) Score scatter plot from an orthogonal partial least squares‐discriminant analysis (OPLS‐DA) that shows the distinct fecal microbiota profile between fecal samples from healthy subjects and IBS patients. (C) A PCA based on the 9699 spectral features detected in fecal supernatants. (D) Score scatter plot from an OPLS‐DA using a VIP cut‐off >1.73 that included the 200 most discriminatory spectral features differentiating the fecal supernatants of healthy subjects and IBS patients. HEALTHY, fecal supernatants from healthy subjects; IBS, fecal supernatants from IBS patients

The microbiota profile of the fecal samples used for preparing fecal supernatants differed between healthy subjects and IBS patients (Figure 6A,B). The bacterial taxa contributing most to the separation were Proteobacteria, *Pseudomonas* spp., *Dorea* spp. and *Ruminococcus gnavus* (*p *< 0.05), and were more abundant in IBS patients, whereas *Bacteroides pectinophilus* was more abundant in healthy subjects (*p *< 0.05). Additionally, the fecal supernatants from healthy subjects and IBS patients, respectively, presented distinct profiles based on the 9699 spectral features detected in an untargeted LC‐MS analysis (Figure 6C). Two hundred of the detected compounds contributed to the separation between the two groups (Figure 6D). Among the top five metabolites in either direction contributing most to the separation, only xanthine has been annotated and was more abundant in fecal supernatants from IBS patients compared with healthy subjects. However, neither metabolite nor microbiota profiles were influenced by the age or sex of the donors (Figure S2).

## 4 DISCUSSION

In this work we describe that components of fecal supernatants, a proxy for the intestinal microenvironment, regulate gene expression of colonoids. Hence, the microbiota and metabolite profiles of fecal supernatants from healthy subjects and IBS patients with predominant diarrhea differed substantially and induced distinct regulation of colonoid gene expression. This suggests that our experimental set‐up, adding patient‐derived fecal supernatants to colonoids established from a healthy donor, has the potential to facilitate studies of intestinal barrier cross talk and explore the impact of the intestinal microenvironment on the pathophysiology of IBS.

Although the recently developed approach of establishing colonoids from intestinal tissue from the individual of interest to create subject‐specific intestinal epithelium has provided a multicellular model system, the importance of luminal factors has been overlooked.^32^ In this study, we therefore exposed colonoids established from a healthy subject to patient‐derived fecal supernatants, to determine effects of luminal factors on the colonic epithelium, potentially involved in IBS pathophysiology. Similar to the commonly used CaCo‐2 cell platform that comes from a patient with colorectal adenocarcinoma, a single healthy donor of colonic tissue was used for this study. Whereas subject‐specific organoids are costly concerning both time and money, collection of fecal samples are non‐invasive and easy to obtain and allow us to investigate the importance of the intestinal microenvironment in IBS versus health. The colonoid monolayers in our study presented with similar features, that is, polarization and specialized cells as mucin‐producing goblet cells, to those previously described in the literature.^27^, ^33^, ^34^, ^35^ The addition of fecal supernatants from IBS patients and healthy subjects induced different gene regulation of the colonoid monolayers, suggesting that the fecal supernatants reproduce the intestinal microenvironment of the donor who provided the fecal material. Importantly, sex or age of donors of fecal supernatants did not influence our results.

The vast majority of genes explored in our study, related to antibacterial response, inflammation and cell junctions, were more highly expressed by the colonoid monolayers stimulated with fecal supernatants from IBS patients compared to healthy subjects. The pro‐inflammatory cytokines IL‐1β^36^ and TNF‐α,^37^ the receptor TLR9 sensing unmethylated CpG dinucleotides of microbial DNA,^38^ the antibacterial enzyme lysozyme^39^ and transcription factors for type 1 interferons IRF5 and IRF7^38^ reflect activation of the immune response and were all expressed to a higher degree in IBS patients. Interestingly, fecal supernatants from IBS patients also induced higher gene expression of CD1D, which encodes an MHC class I‐like molecule presenting bacterial lipid antigens to natural killer T cells (NKT cells). Activated NKT cells rapidly secrete a plethora of cytokines aiming to direct other immune cells to fight infections.^40^ Also, gene expression of TNFSF13 (known as APRIL),^41^ a cytokine supporting class switching to IgA, was higher in colonoid monolayers stimulated with fecal supernatants from IBS patients. Altogether, this suggests that fecal supernatants from IBS patients induce immune activity of epithelial cells. While an impaired barrier function is considered to be central for IBS pathophysiology, it is somewhat surprising to note a higher gene expression of TJP2, DSC2 and CLDN15, known to be involved in maintaining epithelial integrity,^42^ in colonoid monolayers stimulated with fecal supernatants from IBS patients. Our findings are not yet corroborated by other studies, potentially due to the fact that only a few previous studies have addressed the effects of luminal factors on intestinal epithelium, represented by cell lines, organoids or biopsies, in relation to IBS.^13^, ^14^, ^43^ Still, soluble mediators from colonic biopsies from patients decreased the gene expression of ZO‐1, but not occludin, both important components of tight junctions, and induced increased the permeability in Caco‐2 cultures.^14^ Further, fecal supernatants from IBS patients increased the paracellular permeability, assessed by FITC‐Dextran flux, in colonoid cultures.^43^ In parallel, the increase of paracellular permeability in enteroid cultures following stimulation with the proinflammatory stimuli IFN‐γ corresponded with a decrease of the gene expression of the tight junctions ZO‐1 and occludin in the same study.^43^ Similarly, another study provided evidence of impaired barrier function when adding fecal supernatants from IBS patients to intestinal biopsies mounted in Ussing chambers.^13^ In the above mentioned studies, cell function and gene expression was dependent on the type of stimuli^14^, ^43^ as well as concentration and time,^43^ indicating the complexity of studying regulation of barrier function. Hence, the contradictory results may depend on different culture conditions, including concentration of fecal supernatants and time of culture, but also on the different cell systems or tissues. Furthermore, the increased gene expression of junctional complexes recorded in our study may reflect an attempt to regulate expression of tight junction proteins needed to maintain epithelial integrity under IBS‐like conditions. Whether addition of fecal supernatants from IBS patients and healthy subjects, respectively, gives rise to functional differences related to epithelial integrity of the colonoids in our model system remains to be further elucidated. Still, our results support the notion that fecal supernatants from IBS patients have a distinct effect on the intestinal epithelium, regulating genes maintaining epithelial barrier integrity.

In line with a previous study,^14^ fecal supernatants were added to both the apical and basolateral side of the colonoid monolayer to rule out potential effects of not fully confluent monolayers and ensure similar conditions during the stimulations of all the parallel colonoid cultures. Further, it is well‐described that the intestinal epithelial barrier is permeable and certain luminal factors can be transported through the barrier (e.g., intra‐ or paracellularly)^44^ and might reach the basolateral side. Similar to previous studies, we identified a polarized response of the epithelial cells,^18^, ^45^, ^46^ and pro‐inflammatory cytokines were primarily secreted in the apical compartment of the cell cultures. Thus, despite the lack of transepithelial electrical resistance (TEER) measurement, the polarized response of the epithelial cells shows that the monolayers were indeed functional and differentiated and not disrupted by the addition of fecal supernatant or LPS on both sides. There were no differences in the secretion of pro‐inflammatory cytokines between colonoid monolayers stimulated with fecal supernatants from healthy subjects and IBS patients. Interestingly, fecal supernatants had a suppressive effect on cytokine secretion of colonoid monolayers as compared to PBS (negative control) and LPS (positive control). This is in line with a previous study of our group, where macrophages showed reduced cytokine production after conditioning with fecal supernatants as compared to untreated cells.^47^ Altogether, our results sustain the concept of the intestinal microenvironment educating the host to stay unresponsive to the load of bacterial stimuli constantly present in the gut to maintain homeostasis.^47^

The fecal microbiota profiles differed between IBS patients and healthy subjects. The taxa Proteobacteria, *Pseudomonas* spp., *Dorea* spp. and *Bacteroides pectinophilus* were found to be linked to IBS patients in our study, have previously been associated with IBS‐D and symptoms, including intestinal permeability.^48^, ^49^, ^50^, ^51^, ^52^, ^53^, ^54^ Further, the mucin degrader *Ruminoccocus gnavus* has been associated to IBS severity.^55^, ^56^ An altered microbiota composition may result in altered metabolite composition, reflecting the overall function and metabolism of the bacterial community. Indeed, similar to previous reports by us and others, in this study IBS patients presented with distinct fecal metabolite profiles, which were, however, not influenced by age or sex.^9^, ^10^, ^11^ Analyzing the fecal supernatants with LC‐MS allowed us to determine the presence of almost 10,000 spectral features, of which 200 were found significant for separation of IBS from the healthy controls. The possibility to identify compounds from untargeted LC‐MS analysis is, however, resource intensive and the ability to, with reasonable certainty, annotate the top metabolites driving the separation between study groups was limited with the available resources in this project. The only unique annotated top metabolite, the purine‐derived metabolite xanthine, previously demonstrated to promote intestinal barrier development,^57^ was found in higher levels in IBS patients, similar to a previous study by our group.^11^ Besides, although the vast majority of metabolite compounds have yet to be annotated, it is still more likely that the combination of compounds rather than the presence or absence of specific compounds give rise to the IBS‐ specific gene regulation of colonoid monolayers. IBS patients have demonstrated higher concentration of fecal proteases,^13^ which may cause alterations of the epithelial permeability in Caco‐2 cells^12^ and humanized mice.^13^ Additionally, LPS content of fecal samples has been reported to be higher in IBS patients than in healthy subjects.^58^ However, the different effect on gene regulation of colonoid monolayers cultured with fecal supernatants from IBS patients and healthy subjects seen in our study was not driven by LPS, as the level of LPS did not differ between the two groups. Altogether, our data strongly implies that the gene expression profile of colonoid monolayers cultured in the presence of fecal supernatants derived from IBS patients is driven by the content of disease specific luminal factors, with low likelihood of influence of sex hormones or age.

This study has several weaknesses and strengths. The described results originate from experiments using colonoids established from one unique donor and a limited number of study subjects providing fecal supernatants. The time the colonoid monolayers were exposed to fecal supernatants was optimized for detecting regulation of gene expression, which might have jeopardized the possibility to optimally determine secreted cytokines. Also, future experiments to evaluate response to apical exposure alone will clarify potential confounding effect of double‐side stimulation. In addition, factors such as concentration of fecal supernatants, group size or the cytokines measured may have influenced the lack of differences in cytokine secretion. Additionally, this study did not investigate the potential effect of luminal factors on barrier permeability, and analyzing epithelial permeability, measuring for example the TEER, could further have clarified the effect of fecal supernatants on epithelial barrier function, not only gene expression. Moreover, comparing the gene expression of fecal supernatant stimulated organoids to that of colonic mucosal biopsies from IBS and healthy donors could have provided additional validity to our in vitro model. Nevertheless, the study subjects providing fecal samples were all well clinically characterized, and the study design enabled a relatively broad analytical approach while exploring effects on gene regulation using custom made gene arrays. Moreover, the establishment of the microbiota and metabolite profiles of the fecal samples used to prepare supernatants strongly supported the distinct effect of fecal components on gene regulation of the colonoid monolayers, despite the fact that the metabolite composition could not be deciphered in any detail. Even so, this unique study can be regarded as a pilot study proposing an innovative experimental setup for investigating cross talk at the intestinal epithelial barrier, although generalization of results should be cautiously done until further support is available.

In conclusion, the stimulation of polarized and differentiated colonoid monolayers with fecal supernatants from healthy subjects and IBS patients with predominant diarrhea induced distinct gene profiles, potentially reflecting the intestinal microenvironment of the donor providing fecal material. Thus, we propose that the model described in this study may be used to study interactions between components of the intestinal environment and the epithelial layer in more depth, providing improved understanding of the pathophysiology of IBS.

## CONFLICT OF INTEREST

CI, SN, JS, SI, OS and MKM do not have conflict of interest to declare. HT has served as advisory board member for Almirall, Allergan, and Shire. MS has received unrestricted research grants from Danone, Ferring Pharmaceuticals and Glycom (now DSM); served as advisory board member for Almirall, Allergan, Albireo, AstraZeneca, Danone, Nestlé, Glycom (now part of DSM), Menarini, and Shire, as well as a speaker for AlfaSigma, Allergan, Almirall, Alimentary Health, Biocodex, Kyowa Kirin, Menarini, Tillotts, Takeda and Shire. LÖ has received a financial support for research by Genetic Analysis AS, Biocodex, Danone Research and AstraZeneca and served as Consultant/Advisory Board member for Genetic Analysis AS, and as a speaker for Biocodex, Ferring Pharmaceuticals, Takeda, AbbVie, and Meda.

## AUTHOR CONTRIBUTIONS

Guarantor of article: Lena Öhman, PhD. CI carried out the experiments, processed biological data, performed analysis and interpreted, prepared visualizations of the data and drafted the manuscript. SN conceptualized the experimental design, established colonoid cultures, carried out the experiments, and drafted parts of the manuscript. JS provided the healthy study subjects and provided intellectual and scientific input on the final version of the manuscript. SI carried out the experiments. OS performed metabolomic analysis of samples and provided intellectual and scientific input on metabolomic statistical analysis. HT collected the study subject materials and provided intellectual and scientific input on the final version of the manuscript. MKM conceptualized the experimental design, processed and interpreted biological data and critically reviewed the manuscript. MS provided intellectual and scientific input, critically reviewed the manuscript and obtained funding. LÖ conceived the original idea, conceptualized the exploratory study design, interpreted the data, drafted the manuscript and obtained funding. CI and SN shared first authorship. All authors have approved the final draft submitted.

## Supporting information

Supplementary Material

Table S1

Fig S1‐S2

Supplementary Material

Supplementary Material

Supplementary Material
