# Supplementary material for: Fecal luminal factors from patients with irritable bowel syndrome induce distinct gene expression of colonoids
Source: Neurogastroenterol Motil. 2022 Apr 29;34(10):e14390. doi: 10.1111/nmo.14390 (PMC9786662; doi:10.1111/nmo.14390)
Supplement: Supplementary file 1 — Supplementary Material [file NMO-34-e14390-s005.docx]

# **SUPPLEMENTARY MATERIAL**

###### *Study subjects and inclusion criteria*

The study cohort included patients with IBS with predominant diarrhea and healthy subjects (18 - 75 years old). Participants were recruited from the functional gastrointestinal disorders outpatient clinic at the Sahlgrenska University Hospital (Gothenburg Sweden) or through advertisements in the local newspaper. The recruited healthy subjects received monetary reimbursement. Patients were diagnosed according to the Rome IV criteria ^1^ and presented with high moderate or severe symptoms (IBS-SSS score ≥ 175).^2^ Moreover, only patients classified as IBS with predominant diarrhea (predominantly presenting with watery stools (scores of 6-7) according to the Bristol Stool Form scale ^3^) were selected. Healthy subjects had no prior or current gastrointestinal diseases. For both study groups, participants presenting with other severe diseases, pregnancy and breastfeeding were excluded. Further, intake of prebiotics, antibiotics, or medication at least 1 month prior to sample collection were reasons of exclusion to avoid interference with the outcome of the study.

1. *List of targeted genes in the custom RT^2^ PCR arrays*

| GENES RELATED TO ANTIBACTERIAL RESPONSE | |
| --- | --- |
| **Gene name** | **Description** |
| BIRC3 | Baculoviral IAP repeat containing 3 |
| CASP1* | Caspase 1, apoptosis-related cysteine peptidase |
| CASP8 | Caspase 8, apoptosis-related cysteine peptidase |
| CCL5* | Chemokine (C-C motif) ligand 5 |
| CD14 | CD14 molecule |
| CXCL1* | Chemokine (C-X-C motif) ligand 1 (melanoma growth stimulating activity, alpha) |
| CXCL2* | Chemokine (C-X-C motif) ligand 2 |
| CXCL8* | Chemokine (C-X-C motif) ligand 8 |
| HSP90AA1 | Heat shock protein 90kDa alpha family class A member 1 |
| IL-1B* | Interleukin 1, beta |
| IL-6*^#^ | Interleukin 6 |
| IL-18 | Interleukin 18 |
| IRF5* | Interferon regulatory factor 5 |
| IRF7 | Interferon regulatory factor 7 |
| LCN2* | Lipocalin 2 |
| LYZ* | Lysozyme |
| MYD88 | Myeloid differentiation primary response 88 |
| NLRP3 | NLR family, pyrin domain containing 3 |
| PYCARD | PYD and CARD domain containing |
| SLPI | Secretory leukocyte peptidase inhibitor |
| TICAM1 | Toll-like receptor adaptor molecule 1 |
| TLR1 | Toll-like receptor 1 |
| TLR2 | Toll-like receptor 2 |
| TLR4 | Toll-like receptor 4 |
| TLR5 | Toll-like receptor 5 |
| TLR6 | Toll-like receptor 6 |
| TLR9 | Toll-like receptor 9 |
| TNF* | Tumor necrosis factor |
| TNFRSF1A | Tumor necrosis factor receptor superfamily, member 1A |
| GENES RELATED TO INFLAMMATORY RESPONSE | |
| **Gene name** | **Description** |
| CASP1* | Caspase 1, apoptosis-related cysteine peptidase |
| CCL2 | Chemokine (C-C motif) ligand 2 |
| CCL5* | Chemokine (C-C motif) ligand 5 |
| CCL20 | Chemokine (C-C motif) ligand 20 |
| CCL25 | Chemokine (C-C motif) ligand 25 |
| CCR9 | Chemokine (C-C motif) receptor 9 |
| CXCL1* | Chemokine (C-X-C motif) ligand 1 (melanoma growth stimulating activity, alpha) |
| CXCL2* | Chemokine (C-X-C motif) ligand 2 |
| CXCL8* | Chemokine (C-X-C motif) ligand 8 |
| CXCL10 | Chemokine (C-X-C motif) ligand 10 |
| CXCL11 | Chemokine (C-X-C motif) ligand 11 |
| CX3CL1 | Chemokine (C-X3-C motif) ligand 1 |
| DEFA6 | Defensin, alpha 6, Paneth cell-specific |
| IL-1B* | Interleukin 1, beta |
| IL-1RN | Interleukin 1 receptor antagonist |
| IL-6*^#^ | Interleukin 6 |
| IL-13 | Interleukin 13 |
| IL-23A | Interleukin 23, alpha subunit p19 |
| IRF5* | Interferon regulatory factor 5 |
| LCN2* | Lipocalin 2 |
| LYZ* | Lysozyme |
| MMP1 | Matrix metallopeptidase 1 (interstitial collagenase) |
| MUC1 | Mucin 1, cell surface associated |
| NOS2 | Nitric oxide synthase 2, inducible |
| PECAM1 | Platelet/endothelial cell adhesion molecule 1 |
| SAA1 | Serum amyloid A1 |
| TFF1 | Trefoil factor 1 |
| TNF* | Tumor necrosis factor |

| GENES RELATED TO EPITHELIAL BARRIER INTEGRITY | | |
| --- | --- | --- |
| **Gene name** | **Description** |  |
| CAV1 | Caveolin 1, caveolae protein, 22kDa |  |
| CDH1 | Cadherin 1, type 1, E-cadherin (epithelial) |  |
| CLDN1 | Claudin 1 |  |
| CLDN2 | Claudin 2 |  |
| CLDN6 | Claudin 6 |  |
| CLDN12 | Claudin 12 |  |
| CLDN15 | Claudin 15 |  |
| DSC2 | Desmocollin 2 |  |
| DSG2 | Desmoglein 2 |  |
| DSP | Desmoplakin |  |
| ESAM | Endothelial cell adhesion molecule |  |
| ICAM1 | Intercellular adhesion molecule 1 |  |
| ICAM2 | Intercellular adhesion molecule 2 |  |
| ITGA2 | Integrin, alpha 2 (CD49B, alpha 2 subunit of VLA-2 receptor) |  |
| JAM2 | Junctional adhesion molecule 2 |  |
| NOTCH2 | Notch 2 |  |
| OCLN | Occludin |  |
| PVRL1 | Poliovirus receptor-related 1 (herpesvirus entry mediator C) |  |
| TJP1 | Tight junction protein 1 |  |
| TJP2 | Tight junction protein 2 |  |
| TJP3 | Tight junction protein 3 |  |

| MISCELLANEOUS; ADDITIONAL GENES | | |
| --- | --- | --- |
| **Gene name** | **Description** | |
| CD1D | CD1d molecule | |
| CHGA | Chromogranin A (parathyroid secretory protein 1) | |
| CHGB^#^ | Chromogranin B | |
| IFNL2^#^ | Interferon, lambda 2; IL-28A | |
| IL-4^#^ | Interleukin 4 | |
| IL-10^#^ | Interleukin 10 | |
| IL-22 | Interleukin 22 | |
| IL-25^#^ | Interleukin 25 | |
| IL-33 | Interleukin 33 | |
| LGR5 | Leucine-rich repeat containing G protein-coupled receptor 5 | |
| REG3G | Regenerating islet-derived 3 gamma | |
| RETNLB^#^ | Resistin like beta | |
| SCG2 | Secretogranin II | |
| TFF3 | Trefoil factor 3 (intestinal) | |
| TLR7^#^ | Toll-like receptor 7^#^ | |
| TLR8^#^ | Toll-like receptor 8 | |
| TNFSF13 | Tumor necrosis factor superfamily member 13 | |
| TPH1^#^ | Tryptophan hydroxylase 1 | |
| TSLP^#^ | Thymic stromal lymphopoietin | |
| **HOUSEKEEPING GENES*** | | |
| **Gene name** | | **Description** |
| ACTB | | Actin, beta |
| B2M | | Beta-2-microglobulin |
| GAPDH | | Glyceraldehyde-3-phosphate dehydrogenase |
| HPRT1 | | Hypoxanthine phosphoribosyltransferase 1 |
| RPLP0 | | Ribosomal protein lateral stalk subunit P0 |

^#^Gene excluded from the analysis (>60% of samples had missing or very high CT values).

*Gene found in more than one category of those presented above.

1. Statistical analysis

Principal component analyses (PCA) were performed using the prcomp-algorithm and visualized using the pca2d-package in R (version 3.6.2). For the PCA, missing values were imputed using the missMDA package. Orthogonal projections least squared-discriminant analyses (OPLS-DA) were performed to visualize and identify correlations between selected Y-variables and X-variables using SIMCA® software (version 15.0.2, MKS Umetrics AB, Umeå, Sweden). The quality of the OPLS-DA was determined by the parameters of R^2^Y and Q^2^, indicating discrimination and predictability, respectively. R^2^Y values ≥ 0.5 correspond to good fit (max. R^2^Y =1). Q^2^ values ≥ 0.4, but no more than 0.3 away than R^2^Y, are considered adequate for biological variables. Variable influence in projection (VIP) cut-off was used when indicated to identify gene candidates with higher contribution to the model. For metabolomic analysis, VIP was applied to reduce the dimensionality of the dataset. All multivariate analysis were performed using Log_10_ -transformed data.

GraphPad Prism (version 7.04, GraphPad Software Inc., San Diego, CA, USA) was used to perform univariate analyses. Categorical variables were compared using Fisher’s Exact test. Continuous variables were compared using Mann-Whitney U test, independent samples t-test (assumed equal variances) or Kruskal-Wallis H test followed by corrected Dunn’s correction for multiple comparison. Data presented in text, figures and tables is shown as mean (SD) or median (interquartile range) or (min - max), depending on the distribution of the data.

References

1. Lacy BE, Mearin F, Chang L, et al. Bowel Disorders. *Gastroenterology*. 2016;150(6):1393-1407.e5. doi:10.1053/j.gastro.2016.02.031

2. Francis CY, Morris J, Whorwell PJ. The irritable bowel severity scoring system: a simple method of monitoring irritable bowel syndrome and its progress. *Aliment Pharmacol Ther*. 1997;11(2):395-402. doi:10.1046/j.1365-2036.1997.142318000.x

3. Heaton KW, O'Donnell LJ. An office guide to whole-gut transit time. Patients' recollection of their stool form. *J Clin Gastroenterol*. 1994;19(1):28-30. doi:10.1097/00004836-199407000-00008
