# Supplementary material for: Fecal luminal factors from patients with irritable bowel syndrome induce distinct gene expression of colonoids
Source: Neurogastroenterol Motil. 2022 Apr 29;34(10):e14390. doi: 10.1111/nmo.14390 (PMC9786662; doi:10.1111/nmo.14390)
Supplement: Supplementary file 2 — Table S1 [file NMO-34-e14390-s001.docx]

**Table S1.** List of the genes (n = 27) contributing to the distinct gene expression profiles of colonoid monolayers stimulated with fecal supernatants from healthy subjects, LPS or PBS.

| **Gene**  **(2^-ΔCT^)** | **HEALTHY**  **(n = 7)** | **LPS**  **(n = 3)** | **PBS**  **(n = 4)** | **P value**  **(HEALTHY vs. LPS vs. PBS)** |
| --- | --- | --- | --- | --- |
| CAV1 | 0.03 (0.02 - 0.04) | 0.03 (0.03 - 0.03) | 0.04 (0.03 - 0.05) | .16 |
| **CCL20** | 0.20 (0.17 - 0.31) | 0.33 (0.32 - 0.36) | 0.33 (0.28 - 0.35) | **.008** |
| CCL25 | 5.3∙10^-4^ (1.3∙10^-5^ - 0.002) | 3.0∙10^-6^ (2.7∙10^-6^ - 3.8 ∙10^-6^) | 4.8∙10^-4^ (8.6∙10^-6^ - 0.004) | .05 |
| CDH1 | 0.99 (0.65 - 1.07) | 1.22 (0.99 - 1.34) | 1.17 (1.00 - 1.28) | .23 |
| CD1D | 0.007 (0.006 - 0.009) | 0.01 (0.008 - 0.01) | 0.006 (0.006 - 0.01) | .13 |
| CXCL11 | 0.004 (0.004 - 0.006) | 0.004 (0.002 - 0.004) | 0.004 (0.003 - 0.004) | .13 |
| CX3CL1 | 0.001 (7.8∙10^-4^ - 0.001) | 0.002 (0.002 - 0.004) | 9.9∙10^-4^ (6.5∙10^-4^ - 0.002) | .07 |
| DEFA6 | 3.4∙10^-4^ (3.1∙10^-5^ - 4.5∙10^-4^) | 3.6∙10^-5^ (1.1∙10^-5^ - 6.7∙10^-5^) | 1.5∙10^-4^(8.5∙10^-5^-1.6∙10^-4^) | .20 |
| **DSC2** | **0.50 (0.31 - 0.61)*** | 0.75 (0.64 - 0.77) | 0.58 (0.50 - 0.66) | **.03** |
| DSG2 | 0.61 (0.59 - 0.78) | 0.58 (0.46 - 0.66) | 0.84 (0.61 - 1.02) | .31 |
| **ICAM1** | 0.017 (0.017 - 0.019) | 0.03 (0.02 - 0.03) | 0.018 (0.016 - 0.02) | **.04** |
| ICAM2 | 0.006 (0.005 - 0.008) | 0.006 (0.005 - 0.008) | 0.004 (0.004 - 0.007) | .20 |
| IL-1β | 8.7∙10^-4^ (4.3∙10^-4^ - 0.001) | 0.001 (8.5∙10^-4^ - 0.002) | 0.002 (0.001 - 0.002) | .08 |
| IL-33 | 0.002 (0.001 - 0.005) | 5.5∙10^-4^ (4.8∙10^-4^-7.4∙10^-4^) | 0.002 (7∙10^-4^ - 0.002) | .06 |
| ITAG2 | 0.07 (0.05 -0.12) | 0.12 (0.10 - 0.14) | 0.12 (0.10 - 0.14) | .30 |
| IRF7 | 0.004 (0.003 - 0.006) | 0.01 (0.006 - 0.01) | 0.004 (0.003 - 0.006) | .10 |
| MMP1 | 0.17 (0.13 - 0.20) | 0.17 (0.17 -0.21) | 0.28 (0.20 - 0.37) | .14 |
| NOTCH2 | 0.017 (0.015 - 0.02) | 0.012 (0.01 - 0.02) | 0.02 (0.01 - 0.02) | .14 |
| **PECAM1** | **2.0∙10^-4^(1.8∙10^-4^ -5.4∙10^-4^)*** | 2.7∙10^-5^ (2.4∙10^-5^ -3∙10^-5^) | 2.8∙10^-4^(8.7∙10^-5^- 4.2∙10^-4^) | **.02** |
| **PVRL1** | **0.006 (0.005 - 0.006)**^±^ | 0.005 (0.005 - 0.006) | 0.004 (0.004 - 0.005) | **.008** |
| TFF1 | 3.39 (2.44 - 3.61) | 2.42 (2.35 - 2.81) | 3.43 (3.05 - 3.80) | .11 |
| TJP1 | 0.15 (0.13-0.23) | 0.15 (0.14 - 0.17) | 0.22 (0.20 - 0.23) | .24 |
| TJP2 | 0.15 (0.10 - 0.17) | 0.18 (0.13 -0.21) | 0.16 (0.15 - 0.19) | .41 |
| TLR6 | 0.001 (9.9∙10^-4^ - 0.002) | 0.0018 (0.0015 - 0.002) | 0.003 (0.002 - 0.003) | .13 |
| TNF | 0.002 (0.0016 -0.003) | 0.004 (0.0037 - 0.005) | 0.0027 (0.0019 - 0.003) | .06 |
| TNFRSF1A | 0.12 (0.07 - 0.13) | 0.18 (0.14 - 0.19) | 0.11 (0.10 - 0.15) | .06 |
| TNFSF13 | 0.0013 (0.0009 - 0.0017) | 0.0024 (0.001 - 0.0026) | 0.001 (0.001 - 0.002) | .36 |

HEALTHY, colonoid monolayers stimulated with fecal supernatants from healthy subjects; LPS, LPS-stimulated colonoid monolayers; PBS, PBS-stimulated colonoid monolayers. 2^-ΔCT^: 2^(‐delta(cycle threshold))^. Data shown as median (25^th^ - 75^th^ percentile). Kruskal‐Wallis U test between groups followed by Dunn’s multiple comparison post hoc test. Significant genes, group and p values are highlighted in **bold**. *****Colonoid monolayers stimulated with fecal supernatants from healthy subjects vs. LPS-stimulated monolayers: P < .05. ^±^Colonoid monolayers stimulated with fecal supernatants from healthy subjects vs. PBS-stimulated colonoid monolayers: P < .05
