# Supplementary material for: Fecal luminal factors from patients with irritable bowel syndrome induce distinct gene expression of colonoids
Source: Neurogastroenterol Motil. 2022 Apr 29;34(10):e14390. doi: 10.1111/nmo.14390 (PMC9786662; doi:10.1111/nmo.14390)
Supplement: Supplementary file 3 — Fig S1‐S2 [file NMO-34-e14390-s006.docx]

**FIGURE S1. Influence of age and sex on the gene expression of colonoid monolayers stimulated with fecal supernatants from healthy subjects or IBS patients.** Colonoid monolayers were stimulated for 24 h with fecal supernatants from healthy subjects (n = 7 grey dots) or IBS patients (n = 9 black dots). Gene expression was analyzed by PCR arrays for genes involved in antibacterial and inflammatory response and barrier function. Principal component analysis (PCA) based on 75 genes (11 unique genes excluded as they were below detection limit), with the (A) age and (B) sex of each subject identified. HEALTHY, fecal supernatants from healthy subjects; IBS, fecal supernatants from IBS patients. Triangles represent men; small circles correspond to women.





**Figure S2.** **Influence of age and sex on the fecal microbiota and metabolite profiles from healthy subjects and irritable bowel syndrome (IBS) patients.** Fecal samples were analyzed by GA-map^TM^ dysbiosis test while fecal supernatants were analyzed by untargeted liquid chromatography/mass spectrometry analysis. (A, B) Principal component analysis (PCA) based on the 54 bacterial taxa determined in fecal samples from healthy subjects (n = 7 grey dots) and IBS (n = 9 black dots), with the (A) age and (B) sex of each subject identified. (C, D) PCA based on the 9,699 features detected in fecal supernatants from healthy subjects and IBS, with the (C) age and (D) sex of each subject identified. HEALTHY, fecal supernatants from healthy subjects; IBS, fecal supernatants from IBS patients. Triangles represent men; small circles correspond to women.
